# Clinical Outcomes With Dabrafenib Plus Trametinib in a Clinical Trial Versus Real-World Standard of Care in Patients With *BRAF*-Mutated Advanced NSCLC

**DOI:** 10.1016/j.jtocrr.2022.100324

**Published:** 2022-04-06

**Authors:** Bruce E. Johnson, Christina S. Baik, Julien Mazieres, Harry J.M. Groen, Barbara Melosky, Jürgen Wolf, Fatemeh Asad Zadeh Vosta Kolaei, Wen-Hsing Wu, Stefanie Knoll, Meryem Ktiouet Dawson, Adam Johns, David Planchard

**Affiliations:** a Dana-Farber Cancer Institute, Harvard Medical School, Boston, Massachusetts, USA; b Fred Hutchinson Cancer Research Center, University of Washington, Seattle, Washington, USA; c Centre Hospitalier Universitaire de Toulouse, Université Paul Sabatier, Toulouse, France; d Department of Pulmonary Diseases, University Medical Center Groningen, Groningen, The Netherlands; e Department of Medicine, University of British Columbia, Vancouver, British Columbia, Canada; f Center for Integrated Oncology, University Hospital of Cologne, Cologne, Germany; g Novartis Pharma AG, Basel, Switzerland; h Genesis Research, Hoboken, New Jersey, USA; i Novartis Pharmaceuticals Corporation, East Hanover, New Jersey, USA; j Thoracic Group, Medical Oncology Department, Gustave Roussy, Villejuif, France

**Keywords:** BRAF inhibitor, MEK inhibitor, *BRAF*-mutated aNSCLC, Indirect comparison, Platinum-based chemotherapy, Immune-checkpoint inhibitors

## Abstract

**Introduction:**

*BRAF* mutations are rare in patients with NSCLC, and treatment options are limited. Dabrafenib plus trametinib (dab-tram) was approved for *BRAF*^V600^-mutated advanced NSCLC (aNSCLC), based on results from a phase 2 study (NCT01336634). This retrospective analysis compared the effectiveness of dab-tram, based on previously reported clinical trial data, versus real-world standard of care in patients with *BRAF*-mutated aNSCLC.

**Methods:**

Real-world cohorts were derived from a deidentified real-world database (2011–2020) and included patients with *BRAF*-mutated aNSCLC receiving first-line platinum-based chemotherapy (PBC), first-line immune checkpoint inhibitors (ICIs) plus PBC, or second-line ICIs. Weighting by odds was used to estimate the average treatment effect of the treated.

**Results:**

For first-line dab-tram versus PBC, the hazard ratio (HR; 95% confidence interval) for death in unweighted and weighted analyses was 0.65 (0.39–1.1) and 0.51 (0.29–0.92; *p* = 0.03), respectively; unweighted and weighted median overall survival was 17.3 (12.3–40.2) versus 14.5 (9.2–19.6) months and 17.3 (14.6-not reached) versus 9.7 (6.4–19.6) months, respectively. Hazard ratio of death in unweighted and weighted analyses was 0.56 (0.29–1.1) and 0.57 (0.28–1.17), respectively, with first-line dab-tram versus PBC plus ICI, and 0.65 (0.39–1.07) and not reported (Cox proportional-hazards assumption violated), respectively, with second-line dab-tram versus ICI.

**Conclusions:**

In this indirect comparison in patients with *BRAF*-mutated aNSCLC, the risk of death was lower and median overall survival was longer with first-line dab-tram versus PBC. In analyses of dab-tram versus first-line PBC plus ICI or second-line ICI, sample sizes were small and findings were inconclusive with overlapping confidence intervals. Despite some limitations, the study provides useful data for this rare patient population.

## Introduction

In NSCLC, mutations in *BRAF* are an uncommon form of genomic alteration, with a prevalence of 2% to 5% in lung adenocarcinomas.^1^, ^2^, ^3^ BRAF is a serine-threonine kinase of the RAS-RAF-MEK-ERK signaling cascade, a key pathway of cell proliferation, differentiation, and survival, where it activates its downstream targets MEK1 and MEK2.^4^ A range of different *BRAF* mutations have been detected, of which 45% to 83% are of the *BRAF*^V600E^ subtype.^5^, ^6^, ^7^, ^8^, ^9^ *BRAF* mutations occur mostly in individuals with a smoking history, although *BRAF*^V600^ mutations can be identified in patients who never smoked.^1^^,^^6^^,^^8^

On the basis of practice guidelines from the United States, Canada, and Europe, (National Comprehensive Cancer Network, European Society for Medical Oncology, and American Society of Clinical Oncology/Ontario Health [Cancer Care Ontario]),^3^^,^^10^^,^^11^ the standard of care (SoC) for patients with *EGFR*- and *ALK*-negative NSCLC generally includes platinum-based therapy or immune checkpoint inhibitors (ICIs). Nevertheless, for patients with *BRAF*-mutated advanced NSCLC (aNSCLC), evidence on the efficacy of chemotherapy or ICIs is limited owing to the rarity of this patient population. With first-line platinum-based chemotherapy (PBC), retrospective real-world (RW) studies reported similar outcomes in *BRAF*-mutated patients as in those with wild-type tumors (response rates of 50% and 48%, respectively),^2^ except in those with the *BRAF*^V600E^ subtype who seem to be less responsive to PBC (response rate, 29%) than those with other *BRAF* mutations.^1^^,^^2^ For ICIs, that is, agents targeting programmed cell death protein-1 or programmed death-ligand 1 (PD-L1), either as monotherapy or in combination with chemotherapy, a therapeutic benefit has been established in NSCLC populations that typically exclude patients with *EGFR* or *ALK* mutations.^12^ However, data on their efficacy in patients with *BRAF*-mutated aNSCLC are limited to small RW studies, which reported response rates of 24% to 35%.^13^, ^14^, ^15^ Thus, the efficacy and safety of ICIs in patients with *BRAF*-mutated NSCLC remain uncertain.

Dabrafenib and trametinib are inhibitors of BRAF and MEK, respectively, and their combination (dabrafenib plus trametinib [dab-tram]) is the only approved treatment for patients with *BRAF*^V600^-mutated aNSCLC.^16^, ^17^, ^18^, ^19^ The clinical utility of dab-tram in *BRAF*^V600E^-mutated aNSCLC has been revealed in a noncomparative open-label, phase 2 trial, which reported overall response rates of 64% in treatment-naive patients,^20^ and 63.2% in pretreated patients,^21^ with a median overall survival (OS) of 17.3 months and 18.2 months,^22^ respectively. Dab-tram was well tolerated and had a safety profile similar to that previously reported in patients with *BRAF*^V600^-mutant metastatic melanoma.^20^, ^21^, ^22^, ^23^, ^24^ On the basis of these findings, dab-tram was approved in the United States for the treatment of patients with *BRAF*^V600E^-mutated aNSCLC, as detected by a Food and Drug Administration–approved test,^16^ and in Canada and the European Union for the treatment of patients with *BRAF*^V600^-mutated NSCLC.^17^^,^^25^ However, there is a lack of comparative evidence on the efficacy of dab-tram versus other conventional therapies for patients with *BRAF*-mutated NSCLC. Because randomized, controlled trials are not always feasible for rare patient populations owing to practical or ethical reasons, indirect comparisons with synthetic control groups based on RW data are increasingly being used and accepted by regulatory bodies if appropriate clinical trial data are not available.^26^^,^^27^

The aim of this study in patients with *BRAF*-mutated aNSCLC was to indirectly compare the effectiveness of dab-tram, based on data from the previously reported clinical trial, versus RW SoC, based on a deidentified, electronic health record (EHR)–derived database.

## Materials and Methods

### Study Design and Patients

This retrospective study included patients with *BRAF*-mutated aNSCLC who were treated with either dab-tram in a phase 2 clinical trial (NCT01336634) or SoC in RW practice. Details of the dab-tram clinical trial have been published previously (see also Supplementary Methods)^20^^,^^21^; the data cutoff date for the present analysis was June 22, 2019.^22^ Data for RW cohorts were abstracted from the deidentified EHR-derived Flatiron Health database and included patients who had been diagnosed with aNSCLC between January 2011 and February 2020. The Flatiron Health database is a longitudinal database, comprising deidentified patient-level, structured and unstructured data, curated by means of technology-enabled abstraction.^28^^,^^29^ During the study period, the deidentified data originated from approximately 280 U.S. cancer clinics (approximately 800 sites of care).^28^^,^^29^

RW patients had advanced disease (stage IIIB, IIIC, IVA, or IVB), initiated RW SoC (i.e., the index treatment; see subsequent texts) in the metastatic setting on or before January 31, 2020 (i.e., the index date), were aged 18 years or older at the index date, and had a *BRAF* mutation in their lung cancer tissue. Because *BRAF*-mutational subtype status was not consistently documented in the Flatiron Health database, RW patients were not limited to *BRAF*^V600E^. Full inclusion and exclusion criteria of RW patients are described in the Supplementary Methods. Patients with missing baseline variables except Eastern Cooperative Oncology Group performance status (ECOG PS) were excluded from the analysis as a concern to the propensity score model. Patients with missing ECOG PS were included and imputed with a value of 1. No other baseline parameters were imputed. The study included the following three treatment comparisons (Table 1): (1) first-line dab-tram versus PBC; (2) first-line dab-tram versus ICI plus PBC; and (3) second-line dab-tram versus ICI. The selection of RW SoC comparator groups was based on recommendations by practice guidelines (National Comprehensive Cancer Network, European Society for Medical Oncology, and American Society of Clinical Oncology/Ontario Health [Cancer Care Ontario]) for the treatment of *EGFR*- and *ALK*-negative NSCLC disease^3^^,^^10^^,^^11^ and the regimen’s frequency of use in the United States and Canada at the time of the study. PD-L1 expression level, a biomarker for ICI therapy (including pembrolizumab monotherapy), was not recorded in the dab-tram clinical trial. Therefore, inclusion of first-line pembrolizumab monotherapy as a comparator group was not feasible (see Supplementary Methods for further details).

**Table 1:** Treatment Comparisons

Comparison	Real-World SoC Treatments
First-line dab-tram vs. PBC	• Carboplatin + pemetrexed • Carboplatin + paclitaxel • Carboplatin + nab-paclitaxel (protein-bound) • Cisplatin + pemetrexed • Cisplatin + etoposide
First-line dab-tram vs. ICI + PBC	• Pembrolizumab + carboplatin + pemetrexed
Second-line dab-tram vs. ICI	• Pembrolizumab • Nivolumab • Atezolizumab • Durvalumab^a^

*Note:* In the first-line setting, the real-world comparator groups included platinum-based chemotherapy to reflect the SoC during the early part of the study period (i.e., before approval of pembrolizumab monotherapy in 2016 and as combination therapy in 2017), and ICI-based regimens, to reflect the SoC during the later parts of the study. Because pembrolizumab was the only ICI approved in the first-line setting during the time of the study, and because PD-L1 biomarker status was not collected in the dab-tram clinical trial, pembrolizumab in combination with chemotherapy, irrespective of PD-L1 expression status, was selected as first-line ICI-based comparator (with pembrolizumab + carboplatin + pemetrexed identified as the most often used pembrolizumab-based chemotherapy combination regimen). In the second-line setting, the real-world comparator group included all available ICI monotherapies, which were combined for analyses, because individual assessment of these agents would have resulted in small sample sizes.

Dab-tram, dabrafenib plus trametinib; ICI, immune checkpoint inhibitor therapy; PBC, platinum-based chemotherapy; PD-L1, programmed death-ligand 1; SoC, standard of care.

a At the time of study, approved for patients with unresectable stage III NSCLC whose disease has not progressed after concurrent PBC and radiation therapy.

### Study End Points

Study end points included OS, progression-free survival (PFS; dab-tram cohorts), and RW PFS (rwPFS; RW cohorts). In the RW setting, death was based on a composite mortality variable.^30^ rwPFS was defined as the time from start of SoC to RW progression of disease (occurring 14 d after the index date) or RW death, whichever came first. RW progression was assessed retrospectively based on data abstracted from EHRs (see Supplementary Methods).

### Patient Weighting

Full details of patient weighting are shown in the Supplementary Methods. In brief, confounding baseline covariates between dab-tram and RW cohorts were adjusted by propensity score–based weighting by odds to estimate the average treatment effect of the treated.^31^^,^^32^ Baseline characteristics (at index date) used as covariates for the logistic regression model to estimate the propensity score were based on their established prognostic or confounding impact and their availability in the RW database, and included age group, sex, ECOG PS baseline score, history of smoking, race, and (for second-line cohorts only) time between initial NSCLC diagnosis and index date. Each dab-tram patient was assigned a weight of 1, and each RW patient was assigned a weight proportional to their odds of being in the respective dab-tram cohort. Weighted sample sizes were calculated by summing all weighted patients per cohort (i.e., sum of all patients, with each patient multiplied by their individual weight). Standardized mean differences (SMDs) between dab-tram and RW cohorts before and after weighting were summarized, with an SMD of less than 0.25 considered to be indicative of balanced cohorts.

### Statistical Analysis

Time-to-event analyses for OS, PFS and rwPFS were performed using Kaplan–Meier (KM) analyses. A Cox proportional-hazards model was fitted to estimate the hazard ratio (HR) of an event with dab-tram versus SoC. The proportional-hazard assumption was tested through model-based diagnostics, such as including a time-dependent explanatory variable in the model and by visual inspection of the KM plots. For comparisons where the proportional-hazards assumption was violated, HR results were not reported (NR). In addition, the median point estimates for OS, PFS and rwPFS, with 95% confidence intervals (CIs), were calculated. See the Supplementary Methods for further information.

### Ethics

The dab-tram clinical trial was done in accordance with the provisions of the Declaration of Helsinki and Good Clinical Practice guidelines, and the protocol was approved by the Institutional Review Board at each study site. All patients provided written informed consent. Institutional Review Board approval of the study protocol for data collection from the RW cohort was obtained before study conduct and included a waiver of informed consent.

## Results

### Patient Attrition

Records from 61,094 patients with aNSCLC were available in the RW database during the study period of January 1, 2011, to February 29, 2020. Of these, 16,181 patients (26%) and 2366 patients (4%) had initiated first-line PBC and first-line ICI plus PBC, respectively, on or before January 31, 2020, and 165 (1%) and 59 patients (2%), respectively, also had evidence of a positive *BRAF* mutation (Supplementary Table 1). After applying additional inclusion and exclusion criteria, 64 and 34 patients with *BRAF*-mutated aNSCLC were eligible for inclusion in the first-line PBC and ICI plus PBC cohorts, respectively. In the second-line setting, 6214 patients (10%) had initiated second-line ICI monotherapy on or before January 31, 2020, 122 patients (2%) also had evidence of a positive *BRAF* mutation, and 42 patients fulfilled all inclusion and exclusion criteria of the second-line ICI monotherapy cohort (Supplementary Table 2). For dab-tram cohorts, all 36 treatment-naive patients and all 57 pretreated patients (consisting of 38 patients on second-line therapy and 19 patients on third-line therapy or later) who had received dab-tram in the clinical trial were included in the first-line and second-line dab-tram cohorts, respectively.^20^^,^^21^

### Sample Sizes and Patient Characteristics Before and After Weighting

Before weighting, RW cohorts included 64 patients (PBC), 34 patients (ICI + PBC), and 42 patients (ICI), respectively. Because RW patients could be assigned a weight of less than 1 or more than 1 (Supplementary Fig. 1*A*–*C*), the sample sizes of weighted RW cohorts, that is, the sum of all weighted patients, could increase or decrease. Thus, after weighting, the sample sizes of weighted RW cohorts were 37 (PBC), 28 (ICI + PBC), and 54 (ICI), respectively. In the ICI plus PBC cohort, 1 patient with a disproportionately high weight (9.1; approximately, 8.6-times the value of the expected weight for this cohort; Supplementary Fig. 1*B*) was excluded (trimmed) from this cohort to avoid variability in the treatment effect owing to extreme weights. This patient did not have any events of RW progression or RW death over 14 months of follow-up from the start of the treatment. Dab-tram patients were all assigned a weight of 1, and thus, the sample sizes of the dab-tram cohorts remained the same before and after weighting (first-line, 36; second-line, 57).

Baseline patient characteristics before and after weighting are found in Tables 2 and 3. After weighting, baseline characteristics were balanced between the first-line dab-tram and PBC cohorts and between the second-line dab-tram and ICI cohorts (Supplementary Fig. 2*A* and *D*), with an SMD less than 0.25 for all observed covariates. For the first-line dab-tram and ICI plus PBC cohorts, some covariates (smoking history and sex) remained unbalanced even after weighting and trimming (Supplementary Fig. 2*B* and *C*). Across all cohorts, patients initiated their treatment after 2013, with all dab-tram patients treated from 2013 to 2015 and most SoC patients treated from 2016 to 2019.

**Table 2:** Patient Characteristics in First-Line Cohorts Before and After Weighting by Odds

Characteristic	First-Line Dab-Tram (n = 36)	First-Line PBC		First-Line ICI + PBC	
		Preweighting (n = 64)	Weighted (n = 37)	Preweighting (n = 34)	Weighted (n = 28)
Sex, n (%)					
Female	22 (61)	35 (55)	21 (58)	14 (41)	13 (45)
Male	14 (39)	29 (45)	16 (43)	20 (59)	15 (55)
Age at index date^a^					
Mean (SD)	68 (11)	67 (9)	68 (7)	70 (8)	68 (8)
Median	67	66.5	70	70	67
Range	44–91	47–82	47–82	46–83	46–83
Race, n (%)^b^					
White	30 (83)	49 (77)	32 (86)	27 (79)	24 (85)
Black or African	1 (3)	6 (9)	1 (3)	3 (9)	2 (8)
Asian	3 (8)	2 (3)	1 (3)	0 (0)	0 (0)
Other or unknown	2 (6)	7 (11)	3 (8)	4 (12)	2 (7)
Country, n (%)^c^					
USA	9 (25)	64 (100)	37 (100)	34 (0)	28 (100)
Other	27 (75)	0 (0)	0 (0)	0 (0)	0 (0)
Smoking status, n (%)^d^					
Former or current smoker	26 (72)	58 (91)	26 (70)	32 (94)	25 (90)
Nonsmoker	10 (28)	6 (9)	11 (30)	2 (6)	3 (10)
ECOG PS at baseline, n (%)					
0	13 (36)	16 (25)	14 (37)	10 (29)	11 (38)
1	22 (61)	41 (64)^e^	22 (60)	16 (47)^f^	16 (58)
2	1 (3)	7 (11)	1 (3)	8 (24)	1 (3)
Treatment, n (%)					
Dab + tram	36 (100)	0	0	0	0
Carboplatin, pembrolizumab, pemetrexed	0	0	0	34 (100)	28 (100)
Carboplatin-based chemotherapy	0	58 (91)	33 (90)	0	0
Cisplatin-based chemotherapy	0	6 (9)	3.9 (11)	0	0
Time from initial diagnosis to treatment, mo^g^					
Median	2.0	1.5	1.5	1.4	1.9
Range	1.0–63.2	0.5–44.7	0.5–44.6	0.4–124.6	0.4–124.6
Duration of index treatment (first-line), mo^h^					
Median	10.1	1.9	1.4	5.8	6.9
Range	0.3–62.2	0–8.3	0–8.3	0–24	0–24
Q1–Q3	3.2–28.8	1.1–3.1	0.8–2.8	2.1–10.6	2.8–11.7
Treatment initiation y, n %					
2013–2015	36 (100)	20 (31)	10 (28)	0	0
2016–2019	0	44 (69)	26 (72)	34 (100)	28 (100)

ATT, average treatment effect of the treated; dab-tram, dabrafenib plus trametinib; ECOG PS, Eastern Cooperative Oncology Group performance status; ICI, immune checkpoint inhibitor therapy; PBC, platinum-based chemotherapy; Q1, first quartile; Q3, third quartile; USA, United States of America.

a Only birth year was available in Flatiron Health database age was calculated as the number of years between birth year and year of treatment start. Age at enrollment was available for the dab-tram cohort.

b In the weighting analysis, race was recategorized as “White” versus “other,” with “other” including Black, African, Asian, other, and unknown.

c The dab-tram clinical trial was a global trial.

d Smoking history was abstracted from electronic health records and was documented as a history of smoking or no history of smoking; no patient had unknown smoking history.

e A total of 14 of 64 patients (22%) had ECOG PS imputed to 1.

f A total of 6 of 34 patients (18%) had ECOG PS imputed to 1.

g For dab-tram cohorts, the date of diagnosis was derived using the “time since first diagnosis” variable in the trial data. For patients with missing values for this variable, time since diagnosis was imputed with the median value for these patients when deriving weights using the ATT methodology.

h Treatment duration in real-world cohorts was defined as time from the start date of the treatment to the last drug episode date within the same treatment included in the Flatiron drug episode data set.

**Table 3:** Patient Characteristics in Second-Line Cohorts Before and After the Weighting by Odds

Characteristic	Second-Line Dab-Tram (n = 57)	Second-Line ICI	
		Preweighting (n = 42)	Weighted (n = 54)
Sex, n (%)			
Female	28 (49)	23 (55)	30 (55)
Male	29 (51)	19 (45)	25 (45)
Age at index date^a^			
Mean (SD)	65 (10)	68 (8)	65 (10)
Median	64	69	62
Range	41–88	50–82	50–82
Race, n (%)^b^			
White	49 (86)	33 (79)	46 (85)
Black or African	2 (4)	1 (2)	1 (1)
Asian	4 (7)	1 (2)	2 (3)
Other or unknown	2 (4)	7 (17)	6 (11)
Country, n (%)^c^			
USA	14 (25)	42 (100)	54 (100)
Other	43 (75)	0 (0)	0 (0)
Smoking status, n (%)^d^			
Former or current smoking history	41 (72)	35 (83)	41 (76)
Never smoking history	16 (28)	7 (17)	13 (24)
ECOG PS at baseline, n (%)			
0	17 (30)	5 (12)	15 (27)
1	35 (61)	31 (74)^e^	35 (64)
2	5 (9)	6 (14)	5 (9)
Treatment given, n (%)			
Dab + tram	57 (100)	0	0
Atezolizumab	0	1 (2)	1 (2)
Durvalumab^f^	0	3 (7)	4 (6)
Nivolumab	0	30 (71)	40 (74)
Pembrolizumab	0	8 (19)	10 (18)
Time from most recent progression to index date, mo			
Median	1.2	0.6	1
Range	0.1–14.7	0.1–3.9	0–4
Duration of index treatment (second-line), mo^g^			
Median	10.6	3.2^h^	3.7
Range	0.3–61.6	0–57.1	0–57.1
Q1–Q3	4.2–26.6	1.4–10.8	1.3–11.7
Treatment initiation y, n %			
2013–2015	57 (100)	3 (7)	6 (12)
2016–2019	0	39 (93)	48 (88)

dab-tram, dabrafenib plus trametinib; ECOG PS, Eastern Cooperative Oncology Group performance status; ICI, immune checkpoint inhibitor therapy; Q1, first quartile; Q3, third quartile; USA, United States of America.

a Only birth year was available in Flatiron Health database; age was calculated as the number of years between birth year and year of treatment start. Age at enrollment was available for the dab-tram cohort.

b In the weighting analysis, race was recategorized as “White” versus “other,” with “other” including Black, African, Asian, other, and unknown.

c The dab-tram clinical trial was a global trial.

d Smoking history was abstracted from electronic health records and was documented as a history of smoking or no history of smoking; no patient had unknown smoking history.

e A total of 12 of 42 patients (29%) had ECOG PS imputed to 1.

f At the time of study, approved for patients with unresectable stage III NSCLC whose disease has not progressed after concurrent PBC and radiation therapy.

g Treatment duration in real-world cohorts was defined as time from the start date of the treatment to the last drug episode date within the same treatment included in the Flatiron drug episode data set.

h Among patients with >1 year second-line ICI treatment (7 of 42), the median treatment duration was 19.2 months.

In the first-line setting, carboplatin-based chemotherapy was the most common first-line PBC regimen (approximately 90%), and per study protocol, pembrolizumab plus carboplatin and pemetrexed was the only first-line ICI plus PBC regimen. The median treatment duration was longer with first-line dab-tram (10.1 mo) than with first-line PBC (weighted = 1.4 mo (interquartile range quartile 1–quartile 3: 0.8–2.8 mo) and first-line ICI plus PBC (weighted, 6.9 mo). In the second-line ICI setting, nivolumab (approximately 75%) and pembrolizumab (approximately 20%) were the most often used regimens. The median treatment duration was 10.6 months with second-line dab-tram and 3.7 months (weighted) with second-line ICI. Among patients with documented progression, subsequent line of treatment after progression was received by 47.2% of patients (17 of 36) in the first-line dab-tram group, 81.6% of patients (31 of 38) in the first-line PBC group, 63.6% of patients (7 of 11) in the first-line ICI plus PBC group, 54.3% of patients (31 of 57) in the second-line dab-tram group, and 45.5% of patients (10 of 22) in the second-line ICI group (all preweighted cohorts), mostly with chemotherapy-based regimens.

### OS

**Figure 1:**
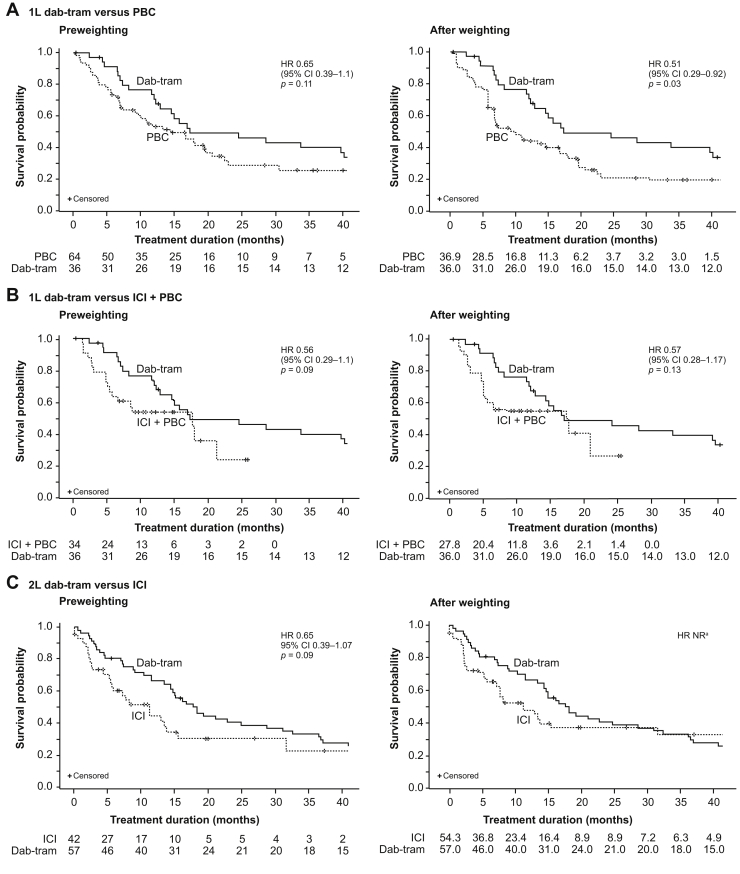
OS with first-line dab-tram versus PBC (A), first-line dab-tram versus ICI + PBC (B), and second-line dab-tram versus ICI (C), both in unweighted (left panels) and weighted (right panels) analyses. Patients who survived through the course of follow-up were censored on the study discontinuation date for dab-tram cohorts and on the date of last encounter in the Flatiron Health database for real-world cohorts. HR was based on a Cox proportional-hazard model with treatment as the primary explanatory variable; *p* values were obtained with Wald chi-square tests with *p* value less than 0.05 considered as statistically significant. ^a^NR because the proportional-hazards assumption was violated. 1L, first line; 2L, second line; CI, confidence interval; dab-tram, dabrafenib plus trametinib; HR, hazard ratio; ICI, immune checkpoint inhibitor; NR, not reported; OS, overall survival; PBC, platinum-based chemotherapy; PD1, programmed cell death protein-1.

In the first-line setting, the HR for death was 0.65 (95% CI: 0.39–1.1, *p* = 0.11) with first-line dab-tram versus RW PBC in the unweighted analysis; however, the risk reduction did not reach statistical significance at the 0.05 level. In the weighted analysis, the risk of death was statistically significantly lower (by 49%) with dab-tram versus PBC (HR = 0.51, 95% CI: 0.29–0.92, *p* = 0.03) (Fig. 1*A*). Likewise, unweighted KM median OS with dab-tram was numerically longer but with overlapping CIs than with RW PBC (17.3 mo [95% CI: 12.3–40.2] versus 14.5 mo [95% CI: 9.2–19.6]) and was statistically significantly longer with dab-tram versus PBC in the weighted analysis (17.3 mo [95% CI: 14.6–NR] versus 9.7 mo [95% CI: 6.4–19.6], difference *p* = 0.01) (Table 4).

**Table 4:** Median OS and PFS

Outcome	First-Line			Second-Line	
	Dab-Tram	PBC	ICI + PBC	Dab-Tram	ICI
Median OS, mo (95% CI)					
Preweighting	17.3 (12.3–40.2)	14.5 (9.2–19.6)	17.7 (5.4–21.3)	18.2 (14.3–28.6)	11.1 (5.4–15.3)
		*p* = 0.11	*p* = 0.09		*p* = 0.09
After weighting	17.3 (14.6–NR)	9.7 (6.4–19.6)	18.0 (5.1–NR)	18.2 (14.3–32.4)	11.2 (5.8–NR)
		*p* = 0.01	*p* = 0.15		*p* = 0.55
Median PFS/rwPFS, mo (95% CI)					
Preweighting	10.2 (5.5–13.8)	4.5 (3.5–5.8)	5.4 (3.7–18.0)	9.7 (5.5–13.6)	3.2 (2.1–8.8)
After weighting	10.2 (7.0–14.5)	4.2 (3.0–5.8)	11.3 (3.7–NR)	9.7 (5.6–13.8)	3.7 (2.1–NR)

*Note:* Median OS were compared between dab-tram and real-world cohorts using 2-sided *p* values from an adjusted log-rank test (after accounting for assigned weights of patients for weighted analyses) at the <0.05 significance level. Median PFS and rwPFS were not statistically compared owing to differences in the definition of disease progression between the dab-tram and real-world cohorts.

dab-tram, dabrafenib plus trametinib; CI, confidence interval; ICI, immune checkpoint inhibitor therapy; NR, not reached; OS, overall survival; PBC, platinum-based chemotherapy; PFS, progression-free survival; rwPFS, real-world progression-free survival.

In the first-line setting using dab-tram and RW ICI plus PBC as control group, the HR of death was 0.56 in the unweighted analysis (95% CI: 0.29–1.1, *p* = 0.09) and 0.57 in the weighted analysis (95% CI: 0.28–1.17, *p* = 0.13), but the risk reduction did not reach statistical significance at the 0.05 level and CIs were wide (Fig. 1*B*). Median OS was similar between dab-tram and ICI plus PBC in both unweighted (17.3 mo [95% CI: 12.3–40.2] versus 17.7 mo [95% CI: 5.4–21.3]) and weighted analyses (17.3 mo [95% CI: 14.6–NR] versus 18.0 mo [95% CI: 5.1–NR]) (Table 4).

In the second-line setting using dab-tram and RW ICI as the control group, the HR of death was 0.65 (95% CI: 0.39–1.07, *p* = 0.09) in the unweighted analysis. In the weighted analysis, the Cox proportional-hazards assumption was violated, as evidenced by crossover of the KM curves, and the HR for death is therefore NR (Fig. 1*C*). Median OS was numerically longer with dab-tram than with RW ICI in the unweighted analysis (18.2 mo [95% CI: 14.3–28.6] versus 11.1 mo [95% CI: 5.4–15.3]) and weighted analyses (18.2 mo [95% CI: 14.3–32.4] versus 11.2 mo [95% CI: 5.8–NR]), but the differences were not statistically significant (Table 4).

### PFS

**Figure 2:**
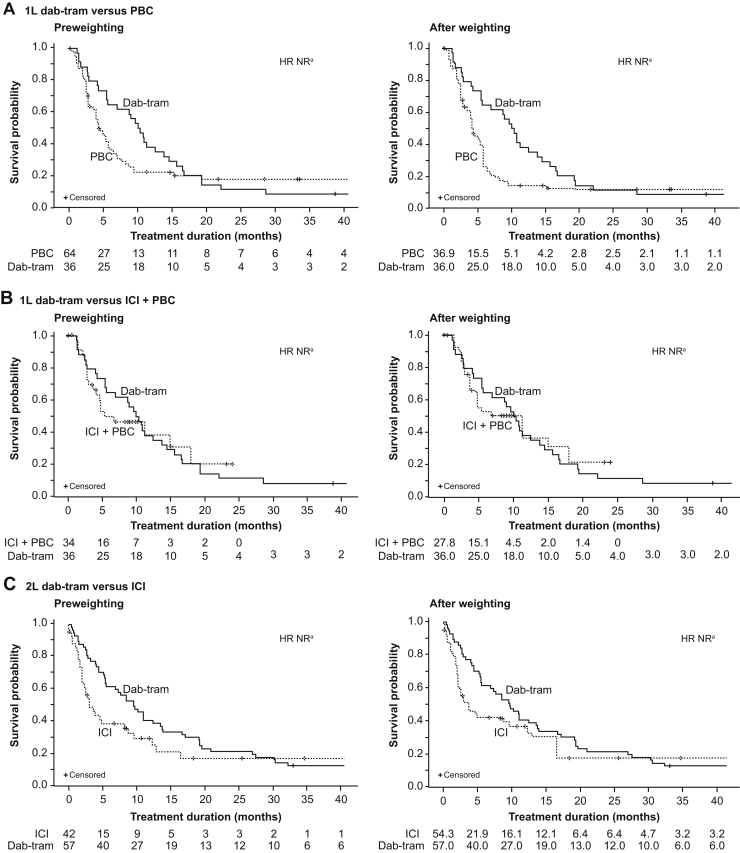
PFS with first-line dab-tram versus PBC (A), first-line dab-tram versus ICI + PBC (B), and second-line dab-tram versus ICI (C), both in unweighted (left panels) and weighted (right panels) analyses. Patients without a death or progression event were censored at their last tumor assessment date for dab-tram cohorts and on the last clinic note date (last date on which progression data were abstracted from physician notes or radiology reports from providers) for real-world cohorts. HR was based on a Cox proportional-hazard model with treatment as the primary explanatory variable. ^a^NR because the proportional-hazards assumption was violated. 1L, first line; 2L, second line; CI, confidence interval; dab-tram, dabrafenib plus trametinib; HR, hazard ratio; ICI, immune checkpoint inhibitor; ICU, intensive care unit; NR, not reported; OS, overall survival; PBC, platinum-based chemotherapy; PFS, progression-free survival.

KM analyses for PFS and rwPFS are illustrated in Figure 2*A*–*C*. With all 3 RW control groups, the proportional-hazards assumption of the Cox regression was violated owing to crossover of the KM curves and HRs are NR. In the first-line setting, median weighted PFS was 10.2 months with dab-tram, whereas weighted rwPFS was 4.2 months with PBC, and 11.3 months with ICI plus PBC. In the second-line setting, median weighted PFS was 9.7 months with dab-tram, and weighted rwPFS was 3.7 months with ICI (Table 4).

## Discussion

For patients with *BRAF*-mutated NSCLC, an uncommon form of lung cancer treatment options is limited and data on their efficacy and safety are scarce. Indirect comparisons represent a valid option to generate contextual information on treatment effectiveness for this patient subgroup, with synthetic RW groups increasingly being used as comparators and accepted by regulatory bodies if appropriate clinical trial data are not available.^26^^,^^27^ In the present study, RW comparator data were from the Flatiron Health database, a longitudinal, EHR-derived, deidentified database comprising patient-level, structured and unstructured data originating from approximately 280 community oncology practices and academic medical centers across the United States of America,^28^^,^^29^ and representing a patient population more clinically diverse than that typically enrolled in clinical trials. The Flatiron Health database has a generally similar demographic and geographic distribution as national registries, such as the Surveillance, Epidemiology, and End Results Program and the National Program of Cancer Registries, and is particularly suited for time-to-event analyses.^28^ In the current study, patients from the dab-tram clinical trial were compared with RW patients using weighting by odds. This method reduces confounding bias, while maintaining all patients in a weighted form in the analysis (except for trimmed cohorts), making it a more reasonable method than other propensity score techniques for balancing patient groups with small sample sizes.^33^^,^^34^

In the first-line setting, dab-tram was assessed in relation to chemotherapy (approximately 90% of carboplatin-based chemotherapy), which before approval of pembrolizumab was the first-line SoC for patients with aNSCLC without *EGFR* or *ALK* mutations and remains a recommended treatment option for patients contraindicated for ICIs.^3^^,^^10^^,^^11^ Relative to RW PBC, dab-tram was associated with a reduced risk of death (by 35%) in the unweighted analysis, which reached statistical significance in the weighted analysis (49% risk reduction; *p* = 0.03). Similarly, weighted median OS with dab-tram was statistically significantly longer than OS with RW PBC (17.3 mo versus 9.7 mo; *p* = 0.01) and was also longer than historic controls of distant lung cancer (no mutation specified) as recorded in the Surveillance, Epidemiology, and End Results Program database (2004–2017; approximately 50% relative survival rate since diagnosis at approximately 10 mo^35^). Median unweighted OS and rwPFS in the present RW PBC cohort were in line with those previously reported in an unadjusted RW cohort study (2009–2012) in U.S. patients with *BRAF*-mutated aNSCLC receiving first-line platinum-based combination chemotherapy (OS = 15.2 mo, PFS = 5.2 mo^3^), supporting the validity of the outcomes observed in the present PBC cohort. Median OS and rwPFS in the present *BRAF*-mutated PBC cohort are also generally similar to survival outcomes observed in clinical trials of first-line PBC in unselected aNSCLC patient populations (median OS = 7.9 mo, median PFS = 3.6 mo^36^). Nevertheless, it has to be noted that the median treatment duration in the first-line PBC cohort was relatively short in relation to median PFS in this cohort, and in relation to the treatment duration in other PBC studies in lung cancer. Nevertheless, overall the findings indicate that in patients with aNSCLC, first-line dab-tram provides a considerable survival benefit, whereas the effect is generally only moderate with first-line PBC.

The second control group in the treatment-naive setting, ICI plus PBC, reflects the shift from chemotherapy to ICI-based SoC during the study period. In the current treatment landscape, recommended first-line treatment options for patients with no actionable *EGFR* or *ALK* alterations include various ICIs, either combined with chemotherapy irrespective of PD-L1 expression status or as monotherapies for patients with PD-L1 greater than or equal to 50%.^3^^,^^10^^,^^11^ Nevertheless, during the time of this study (January 2011–February 2020), pembrolizumab was the only ICI approved in the first-line setting (first approved in 2016 as monotherapy and in 2017 in combination with chemotherapy^37^); because PD-L1 biomarker status was not collected in the dab-tram clinical trial, a comparison with pembrolizumab monotherapy was not feasible. Therefore, pembrolizumab in combination with chemotherapy, irrespective of PD-L1 expression status, was selected as first-line ICI-based comparator group. The risk of death was numerically lower by 43% to 44% with first-line dab-tram versus RW ICI plus PBC, with similar median OS (approximately 17–18 mo), but the risk reduction was not statistically significant, and CIs were wide. However, this analysis is associated with several limitations. First, some covariates remained unbalanced between the dab-tram and ICI plus PBC cohorts even after weighting and trimming of one patient in the ICI plus PBC cohort owing to a disproportionally high weight. Second, follow-up of the ICI plus PBC cohort was immature because the combination of pembrolizumab plus pemetrexed plus carboplatin for first-line treatment had only been approved in the United States since 2017. Hence, RW data for the ICI plus PBC cohort are mostly (>90% of patients) from 2018 and 2019, whereas those for the dab-tram cohort date from 2014 and 2015. Median OS and rwPFS with RW ICI plus PBC in the present study are similar to survival outcomes reported in clinical trials of pembrolizumab + chemotherapy in patients with aNSCLC (nonsquamous metastatic NSCLC: median OS = 22.0 mo, median PFS = 9.0 mo^38^), even though the proportion of patients with *BRAF*-mutated NSCLC in these clinical trials was likely low.

In the second-line setting, dab-tram was assessed in relation to ICI-based monotherapy (approximately 70%–75% of nivolumab, approximately 20% of pembrolizumab). Median unweighted OS and rwPFS observed with RW ICI in the present study are in line with survival outcomes previously reported for RW patients with *BRAF*-mutated aNSCLC receiving ICI monotherapy (median OS = 12.0–22.5,^14^^,^^15^ median PFS = 3.1–5.3 mo,^14^^,^^15^ with 90%–95% patients receiving second-line or later), and with those reported in clinical trials of second-line ICIs in patients with aNSCLC (nivolumab: median OS = 9.2–12.1 mo, median PFS = 2.3–3.5 mo; pembrolizumab: median OS = 10.4–12.7 mo, median PFS = 3.9–4 mo^12^). The weighted HR was NR owing to violation of the model. The risk of death and median OS were numerically lower and longer, respectively, in the unweighted analysis of dab-tram and ICI; however, the observed risk reduction was not statistically significant, and CIs were wide.

More research will be needed to further establish the comparative effectiveness of dab-tram as first-line treatment versus ICIs plus chemotherapy and as second-line treatment versus ICIs for patients with *BRAF*-mutated aNSCLC.

Apart from the caveats mentioned previously, our study is associated with other limitations. Because *BRAF*-mutational subtype status was not reliably reported for all patients in the Flatiron database, the inclusion criterion of *BRAF*^V600E^ mutational subtype status, as specified in the dab-tram trial, could not be applied to the RW groups. Hence, *BRAF* subtypes other than V600E may have been included, which may have affected treatment outcomes.^6^^,^^9^ Nevertheless, based on previous reports, 45% to 83% of patients in our RW cohorts would be expected to be of the *BRAF*^V600E^ subtype.^5^, ^6^, ^7^, ^8^, ^9^ Furthermore, the V600E subtype has been found to be less responsive to PBC than other *BRAF* mutants;^1^^,^^2^ therefore, outcomes in the present PBC cohort may overestimate the treatment effect in *BRAF*^V600E^ patients. Because of the small sample sizes of RW subgroups with recorded *BRAF*-mutational subtype, a subgroup analysis based on *BRAF* subtype would have been of limited value. Likewise, sample sizes were too small for a subgroup analysis by smoking status, which could serve as a proxy for *BRAF*^V600E^ subtype. The proportions of patients with a history of never and former or current smoking in the RW cohorts (weighted) were 10% to 30% and 70% to 90%, respectively, a distribution, which is largely in line with that in the dab-tram cohorts in the present study (28% and 72%, respectively), and with that previously reported for patients of the *BRAF*^V600E^ mutational subtype (22%–48% of never smokers, 52%–78% of former or current smokers).^1^^,^^6^

Results from the PFS and rwPFS analyses have to be interpreted cautiously because of the different criteria used to assess progression in the dab-tram clinical trial and the RW groups. Potential survivorship bias may have been introduced because the time from diagnosis to start of treatment may have been longer for dab-tram than for RW SoC, owing to the time required for *BRAF* genotyping as part of the dab-tram indication. The distribution of *BRAF* mutations may be skewed over time, as panel testing including non-V600E mutations was not available until 2013 to 2015. Nevertheless, whereas the start of study period was 2011, the entire study population initiated their treatment after 2013. Median OS, PFS and rwPFS had generally relatively wide CIs, which is likely related to the small sample sizes of the cohorts resulting from the rarity of the patient population. There was also a relatively large number of censored patients in some RW cohorts relative to the dab-tram cohorts (e.g., first-line PBC + ICI 47% versus dab-tram 39%, second-line ICI 38% versus dab-tram 19%), which could reflect incomplete RW mortality information. Nevertheless, the Flatiron Health mortality composite variable has been validated against the National Disease Index, revealing 83.9% to 91.5% sensitivity and 93.5% to 99.7% specificity for aNSCLC.^30^ It is possible that deaths from the RW setting that occurred close to the end of the study may have been captured after the data cutoff date. Comparisons of OS in the first-line settings may have also been affected by the fact that use of other therapies after progression was more common in RW cohorts than in the dab-tram cohort. Comorbidity, which may have affected treatment outcomes, was not included as a weighting covariate; however, ECOG PS could be considered an indirect measure of comorbidity. Finally, patients from RW cohort could have stage III or IV disease, whereas dab-tram patients generally had stage IV (except for 1 patient in the first-line setting who was stage III at index date).

Other limitations are inherent to the use of RW data. This type of data source has a potential for missing, inaccurate, or incomplete data. Technology-enabled abstraction by specially trained human abstractors using documented procedures and defined quality control activities aim to increase completeness. Nevertheless, ECOG PS information was missing in 18% to 29% of RW patients. For these patients, ECOG PS was imputed to a value of 1, based on Flatiron research indicating patients with NSCLC with missing ECOG PS score have similar outcomes as those with ECOG PS 1. Finally, despite patient weighting, potential selection bias, and unmeasured and residual confounding cannot be fully ruled out. Patients with missing baseline variables except ECOG were excluded from the analysis as a concern to the propensity score model.

Some of the above-mentioned limitations have been partially addressed by another study using RW data, which included RW patients with aNSCLC and a recorded *BRAF*^V600^-mutation, and which compared RW dab-tram versus PBC, ICI plus PBC, or ICI in the first-line setting. In this study, first-line dab-tram was associated with a significant improvement in OS (HR *p* < 0.01, post-weighting) and rwPFS (HR *p* = 0.02, post-weighting) versus first-line PBC, and with numerical improvements in OS versus first-line ICI and versus first-line ICI plus PBC (all weighted analyses), thereby confirming and expanding the findings from the present study.^39^

Despite its limitations, the present study provides additional information by being the first study to provide comparative effectiveness evidence for patients with *BRAF*-mutated aNSCLC, a rare patient population with limited treatment options. The indirect comparison of dab-tram clinical trial data with ICI- and chemotherapy-based RW comparator groups provides contextual evidence on the effectiveness of these treatments, thereby expanding the evidence available from the dab-tram single-arm trial. The RW comparator groups were derived from longitudinal patient-level data from a large RW database in the United States, and patients were balanced for key baseline covariates using established comparative methodology with strict criteria.

Overall, the findings from this indirect comparison of dab-tram clinical trial data versus SoC data from a RW setting suggest that in patients with *BRAF*-mutated aNSCLC, the risk of death is lower and median OS is longer with first-line dab-tram versus PBC, supporting the use of dab-tram versus PBC as a first-line therapy in these patients. In the analyses of dab-tram versus first-line PBC plus ICI or versus second-line ICI, there was a trend toward numerical survival benefits; however, sample sizes were small and findings were inconclusive with overlapping CIs. Thus, further research is needed to establish the comparative effectiveness of these agents in this rare patient population. Despite some limitations, the study provides useful data for this rare patient population.

## CRediT Authorship Contribution Statement

**Bruce E. Johnson:** Conceptualization, Investigation, Interpretation of data, Writing (original draft and review), Supervision.

**Christina S. Baik, Julien Mazieres, Harry J. M. Groen, Barbara Melosky, Jürgen Wolf, David Planchard:** Investigation, Interpretation of data, Writing (original draft and review).

**Fatemeh Asad Zadeh Vosta Kolaei**, **Adam Johns:** Conceptualization, Methodology, Formal analysis, Interpretation of data, Writing (original draft and review), Supervision.

**Wen-Hsing Wu:** Methodology, Formal analysis, Software, Data curation, Visualization, Interpretation of data, Writing (original draft and review).

**Stefanie Knoll:** Conceptualization, Methodology, Interpretation of data, Writing (original draft and review), Supervision.

**Meryem Ktiouet Dawson:** Conceptualization, Interpretation of data, Writing (original draft and review).

## Data-Sharing Statement

Novartis is committed to sharing with qualified external researchers, access to patient-level data, and supporting clinical documents from eligible studies. These requests are reviewed and approved by an independent review panel on the basis of scientific merit. All data provided are anonymized to respect the privacy of patients who have participated in the trial in line with the applicable laws and regulations. The availability of trial data is according to the criteria and process described on www.clinicalstudydatarequest.com. This report contains data originated by Flatiron Health.
