# Supplementary material for: Clinical Outcomes With Dabrafenib Plus Trametinib in a Clinical Trial Versus Real-World Standard of Care in Patients With BRAF-Mutated Advanced NSCLC
Source: JTO Clin Res Rep. 2022 Apr 6;3(5):100324. doi: 10.1016/j.jtocrr.2022.100324 (PMC9112112; doi:10.1016/j.jtocrr.2022.100324)
Supplement: Supplementary Methods [file mmc1.docx]

**Supplemental Digital Content**

**Supplement to:**

**Bruce E. Johnson, Christina S. Baik, Julien Mazieres, Harry J. M. Groen, Barbara Melosky, Jürgen Wolf, Fatemeh Asad Zadeh Vosta Kolaei, Wen-Hsing Wu, Stefanie Knoll, Meryem Ktiouet Dawson, Adam Johns, David Planchard**

**Clinical Outcomes With Dabrafenib Plus Trametinib in a Clinical Trial Versus Real-world Standard of Care in Patients With *BRAF*-Mutated Advanced Non–Small Cell Lung Cancer**

**Supplementary Methods**

### *Study Design and Patients*

Patients in the dab-tram cohorts (ie, in study NCT01336634) were required to be aged ≥18 years, have NSCLC with stage IV disease, have a confirmed *BRAF*^V600E^ mutation (using archived tissue samples; based on local testing in laboratories approved by Clinical Laboratory Improvement Amendments, or equivalent outside the United States), be *EGFR* wild-type and *ALK*-negative, or be *EGFR*-mutated or *ALK*-translocated and with prior *EGFR-* or *ALK*-inhibitor therapy (for first-line cohorts, prior *EGFR-* or *ALK*-inhibitor therapy was only allowed as adjuvant therapy), have ECOG PS 0 to 2, and have measurable disease per Response Evaluation Criteria in Solid Tumors (RECIST) V 1.1. Asymptomatic, untreated, <1 cm, or stable brain metastases were allowed.

Patients in the real-world cohorts, derived from the aNSCLC Flatiron Health database, had a diagnosis of lung cancer (by International Classification of Diseases Coding Manual, 9th or 10th revision), had developed advanced disease (stage IIIB, IIIC, IVA, or IVB) at initial diagnosis on or after January 1, 2011, and had at least 2 documented clinical visits on or after January 1, 2011. In addition, patients were required to have initiated first-line PBC, first-line ICI+PBC, or second-line ICI (ie, the index treatment) in the metastatic setting at a date on or prior to January 31, 2020 (ie, the index date), be aged ≥18 years at the index date, and have a *BRAF*-mutation in lung cancer tissue. Because *BRAF*-mutational subtype status was not consistently captured in Flatiron database, real-world patients were not limited to *BRAF*^V600E^. *BRAF* mutational status had to be known at any time prior to or within a maximum of 60 days after the index date. The maximum 60-day period between index date and *BRAF* test result aimed to minimize potential survival bias in the real-world cohorts, ie, patients who received their *BRAF* test result long after starting treatment would have survived until the time of testing, which could potentially overestimate survival outcomes. Patients were also required to be *EGFR* wild-type and *ALK*-negative, have available progression data, have ECOG PS 0 to 2 within 30 days prior to or 7 days after index date, have adequate organ function within 30 days prior to or 7 days after index date, and have at least 1 visit within 90 days after the date of metastatic NSCLC diagnosis. Patients who met any of the exclusion criteria of the dab-tram clinical trial were excluded from the real-world cohorts.

### *Treatments*

The selection of real-world comparators was based on recommendations by NCCN, ESMO, and ASCO/OH (COC) guidelines for the treatment of *EGFR*- and *ALK*-negative NSCLC disease,^3, 10, 11^ and the availability of regimens in Canada and the US at the time of the study. In the first-line setting, platinum-based chemotherapy was the SoC during the early part of the study period (2011 to ~2017; ie, prior to approval of pembrolizumab monotherapy in 2016 and as combination therapy in 2017), and included carboplatin plus pemetrexed, paclitaxel or nab-paclitaxel (protein-bound), and cisplatin plus pemetrexed or etoposide. The second comparator group in the first-line setting was selected to address the change from chemotherapy to ICI-based regimens during the later parts of the study. Because pembrolizumab was the only ICI agent approved in the first-line setting at the time of the study, and because PD-L1 biomarker status was not collected in the dab-tram clinical trial, a comparison with pembrolizumab monotherapy (approved as first-line therapy in the US and Canada for patients with PD-L1 ≥1% or PD-L1 ≥50%) was not feasible. Therefore, pembrolizumab in combination with chemotherapy, irrespective of PD-L1 expression status, was selected as first-line ICI-based comparator group, and was restricted to pembrolizumab + carboplatin + pemetrexed (identified as the most commonly used pembrolizumab-based chemotherapy combination regimen [Novartis, unpublished data on file]). For the second-line real-world comparator group, all available ICI monotherapies (pembrolizumab, nivolumab, atezolizumab, or durvalumab) were allowed, and were combined for analyses because the individual assessment of these agents would have resulted in small sample sizes.

Dabrafenib (150-mg twice daily) plus trametinib (2-mg once daily) was administered per study protocol.^20, 21^ Treatment was continued until disease progression, death, or unacceptable adverse events, per protocol. Patients with progressive disease were allowed to receive further treatment, either with dab-tram if they had a confirmed response according to RECIST v1.1 or had stable disease lasting ≥12 weeks, or with other therapies. In the real-world cohorts, SoC was administered at the discretion of the treating clinician and according to local practice. real-world patients could receive further treatment after progression, except for BRAF-inhibitors or MEK-inhibitors.

### *Study Endpoints*

### OS was defined as the time of dab-tram or SoW initiation to death. In the real-world setting, death was based on the Flatiron Health mortality composite variable,^30^ a validated, real-world composite mortality endpoint that integrates structured and unstructured data from EHRs, obituary data and the US Social Security Death Index. The Flatiron Health mortality variable reports only the month and year of death, therefore, the date of real-world death was imputed as the 15th of the month. PFS was defined as the time of dab-tram initiation to disease progression or death (whichever came first). Progression was assessed prospectively based on scheduled imaging assessments and RECIST 1.1 criteria (investigator assessed).^20, 21^ Real world PFS (rwPFS) was defined as the time from start of SoC to real-world progression (only progression events that occurred 14 days after initiation of SoC were considered, to account for the potential lag in identifying the date of progression) or death, whichever came first. Real-world progression was assessed retrospectively, based on data abstracted from EHRs, and was defined as a distinct episode, in which the treating clinician concluded that there had been growth or worsening in the disease (based on clinician notes, radiological reports, and pathological reports available in the EHR^40^).

### *Patient Weighting*

### A propensity score-based weighting by odds method was used to adjust for confounding baseline covariates between dab-tram and real-world cohorts and estimate the average treatment effect of the treated (ATT).^31, 32^ The propensity score (PS), representing the probability of receiving dab-tram given a set of observed baseline covariates, was calculated through fitting the logistic regression, and converted to the odds scale (PS/[1 – PS]) to calculate patient weights. Thus, each patient in the dab-tram cohort was assigned a weight of 1, and each real-world patient was assigned a weight proportional to their log odds of being in the respective dab-tram cohort. Baseline characteristics used as covariates for weighting were based on their established prognostic or confounding impact and their availability in the Flatiron database, and included age group, sex, ECOG PS baseline score, history of smoking, race, and (for second-line cohorts only) time between initial NSCLC diagnosis and index date. Patients with missing BL variables except ECOG (see above, study design and patients) were excluded from the analysis as a concern to the propensity score model. Race was recategorized as “White” versus “other,” with “other” including Black, African, Asian, other, and unknown due to small sample sizes. Weighting by odds of receiving treatment retains all patients in the analysis, and may therefore offer the most representative estimate of ATT.^33^ Because the range of weights of reference real-world patients could be <1 or >1, sample sizes of the weighted real-world cohorts (ie, the sum of all weighted patients in this cohort) could increase or decrease to become more similar to the size of the treated (ie, dab-tram) cohort. One real-world patient with an extreme propensity score weight (9.1.) was excluded (trimmed) from the analysis to reduce the chance of bias due to influential observations (ie, over-representation of the patient).^33^ Weight trimming of this patient was based on visual inspection of the propensity score weight distribution (see **Supplementary Figure 1**). Standardized mean differences (SMD) between dab-tram and real-world cohorts before and after weighting were summarized, with an SMD of <0.25 considered to be indicative of balanced cohorts.

### *Statistical Analysis*

No sample size calculation was performed in this study. Baseline characteristics were evaluated before and after patient weighting using descriptive statistics. Time-to-event analyses for OS and PFS/rwPFS were performed using Kaplan-Meier analyses. For OS analyses, patients who survived through the course of follow-up were censored on the study discontinuation date for dab-tram cohorts, and on the date of last encounter in the Flatiron database for real-world cohorts. In PFS and rwPFS analyses, patients without a death or progression event were censored at their last tumor assessment date for dab-tram cohorts, and on the last clinic note date (last date, on which progression data were abstracted from physician notes or radiology reports from providers) for real-world cohorts.

For comparisons of dab-tram vs real-world SoC, a Cox proportional-hazards model was fitted to estimate the hazard ratio (HR) of an event with dab-tram vs real-world SoC (presented with 95% confidence interval [CI]), with treatment as the primary explanatory variable; p-values were obtained with Wald Chi-Square tests with p < 0.05 considered as statistically significant. The proportional-hazard assumption was validated through model-based diagnostics such as including a time-dependent explanatory variable in the model, and by visual inspection of the KM plots. For comparisons where the proportional hazards assumption was violated (ie, where the hazards assumption was violated based on statistical tests or crossover was observed on visual inspection of the KM plots), HR results were not reported. The median point estimates with 95% CIs were also calculated. Median OS were compared using 2-sided p-values from an adjusted log-rank test (after accounting for assigned weights of patients for weighted analyses) at the <0.05 significance level. Median PFS and rwPFS were not statistically compared due to differences in the definition of disease progression between the dab-tram and real-world cohorts. All statistical analyses were performed using the SAS 9.4 software.

**Supplementary Results**

**Supplementary Table 1. Patient Attrition in Real-world first-line PBC and PD1+PBC Cohorts**

|  |  | **First-line PBC** | | **First-line ICI+PBC** | |
| --- | --- | --- | --- | --- | --- |
|  | **Description** | **Number  of patients** | **% patients remaining  from previous step** | **Number  of patients** | **% patients  remaining  from previous step** |
| **1** | Total N in Flatiron advanced NSCLC database^a^ | 61 094 |  | 61 094 |  |
| **2** | Initiated selected regimens as first-line on or prior to January 31, 2020^b^ | 16 181 | 26% | 2366 | 4% |
| **3** | Evidence of positive *BRAF* mutation | 165 | 1% | 59 | 2% |
| **4** | *BRAF* test result was within 60 days of PD1-mono therapy start date^c^ | 110 | 67% | 54 | 92% |
| **5** | Eligible for progression analysis^d^ | 110 | 100% | 54 | 100% |
| **6** | Age ≥18 years at index | 110 | 100% | 54 | 100% |
| **7** | ECOG PS 0, 1 2, or missing^e^ within 30 days prior to index or 7 days after index date | 109 | 99% | 53 | 98% |
| **8** | With adequate organ function 30 days prior to and 7 days after index date^f^ | 67 | 61% | 38 | 72% |
| **9** | At least 1 visit within 90 days after metastatic NSCLC diagnosis date | 67 | 100% | 38 | 100% |
| **10** | Without BRAF-inhibitor or MEK inhibitors any time in the database | 64 | 96% | 35 | 92% |
| **11** | Without *EGFR* mutation or *ALK* rearrangement test with a positive result | 64 | 100% | 35 | 100% |
| **12** | Without evidence of pregnancy at any time in the database | 64 | 100% | 35 | 100% |
| **13** | Eligible for rwPFS and OS analyses ^g^ | 64 | 100% | 34 | 97% |

^a^ Patients with stage IIIB, IIIC, IVA, or IVB NSCLC at diagnosis, or those who presented with earlier stage NSCLC but subsequently developed advanced disease on or after January 1, 2011.

^b^ The last encounter available in the Flatiron Health database was February 29, 2020. Restricting the index date to be no later than January 31, 2020 allowed for at least 30 days of observation time.
^c^ To avoid immortal time bias, ie, patients who had their test result long after treatment start date would have survived up until the time-point of testing, which could potentially overestimate the outcome of interest.
^d^ Only patients, for whom the progression data (the presence or absence of progression) were collected, were included.
^e^ Patients without ECOG PS testing within this period were included. Missing ECOG PS scores were imputed with a value of 1 (based on internal Flatiron research showing that patients with aNSCLC who have missing ECOG PS status generally appear more similar to patients with ECOG PS scores of 1). Among patients included in the final study cohorts, 14 out of 64 patients (22%) in the first-line PBC cohort, and 6 out of 34 patients in the first-line ICI+ PBC cohort (18%) had ECOG PS imputed to 1 (pre-weighted cohorts). 
^f^ Adequate organ function was defined as absolute neutrophil count ≥1.5 × 10^9^/L; hemoglobin ≥9 g/dL; platelet count ≥100 x 10^9^/L; prothrombin time/INRb ≥1.5 x ULN; total bilirubin ≤1.5 x ULN; ALT ≤2.5 x ULN; serum creatinine ≤1.5mg/dL or creatinine clearance ≥50 mL/min. Patients with a missing test result were imputed to have had a normal value.
^g^ Patients with a negative follow-up period were not eligible for rwPFS and OS analyses. Progression information (presence or absence of progression) was based on clinic notes. If the last clinic note date was prior to treatment start, the patient was not eligible for PFS analysis. If the last OS follow-up date was prior to treatment start, the patient was not eligible for OS analysis.

ALT, alanine aminotransferase; ECOG PS, Eastern Cooperative Oncology Group performance status; ICI, immune-checkpoint inhibitor therapy; INRb, international normalized ratio; NSCLC, non–small cell lung cancer; PBC, platinum-doublet therapy; rwPFS, real-world progression-free survival; ULN, upper limit of normal.

**Supplementary Table 2.** Patient Attrition in Real-world second-line ICI Cohort

|  | | **Second-line ICI** | |
| --- | --- | --- | --- |
| **Description** | | **Number  of patients** | **% patients remaining  from previous step** |
| **1** | Total N in Flatiron advanced NSCLC database^a^ | 61 094 |  |
| **2** | Initiated PD(L)1 monotherapy as second-line treatment on or prior to January 31, 2020^b^ | 6214 | 10% |
| **3** | Evidence of positive *BRAF* mutation | 122 | 2% |
| **4** | *BRAF* test result was within 60 days of PD(L)1 monotherapy start date^c^ | 101 | 83% |
| **5** | Received a platinum-based treatment prior to index date | 85 | 84% |
| **6** | Eligible for progression analysis^d^ | 84 | 99% |
| **7** | Age ≥18 years at index | 84 | 100% |
| **8** | ECOG PS 0, 1, 2, or missing^e^ within 30 days prior to index or 7 days after index date | 84 | 100% |
| **9** | With adequate organ function 30 days prior to and 7 days after index date^f^ | 52 | 62% |
| **10** | At least 1 visit within 90 days after metastatic NSCLC diagnosis date | 52 | 100% |
| **11** | Without BRAF-inhibitor or MEK inhibitors any time in the database | 46 | 88% |
| **12** | Without *EGFR* mutation or *ALK* rearrangement test with a positive result | 46 | 100% |
| **13** | Without anticancer therapy within 14 days prior to index date | 42 | 91% |
| **14** | Without evidence of pregnancy at any time in the database | 42 | 100% |
| **15** | Eligible for rwPFS and OS analyses ^g^ | 42 | 100% |
| ^a^ Patients with stage IIIB, IIIC, IVA, or IVB NSCLC at diagnosis, or those who presented with earlier stage NSCLC but subsequently developed advanced disease on or after January 1, 2011. ^b^ The last encounter available in the Flatiron Health database was February 29, 2020. Restricting the index date to be no later than January 31, 2020 allowed for at least 30 days of observation time. | | | |
| ^c^ To avoid immortal time bias, ie, patients who had their test result long after treatment start date would have survived up until the time-point of testing, which could potentially overestimate the outcome of interest. | | | |
| ^d^ Only patients, for whom progression data (the presence or absence of progression) were collected, were included. | | | |
| ^e^ Patients without ECOG PS testing within this period were included. Missing ECOG PS scores were imputed with a value of 1 (based on internal Flatiron research showing that patients with aNSCLC who have missing ECOG PS status generally appear more similar to patients with ECOG PS scores of 1). Among patients included in the final study cohorts, 12 of 42 patients (29%) in the second-line ICI cohort had ECOG PS imputed to 1 (pre-weighted cohort).  ^f^ Adequate organ function was defined as absolute neutrophil count ≥1.5 × 10^9^/L; hemoglobin ≥9 g/dL; platelet count ≥100 x 10^9^/L; prothrombin time/INRb ≥1.5 x ULN; total bilirubin ≤1.5 x ULN; ALT ≤2.5 x ULN; serum creatinine ≤1.5 mg/dL or creatinine clearance ≥50 mL/min. Patients with a missing test result were imputed to have had a normal value. | | | |
| ^g^ Patients with a negative follow-up period were not eligible for rwPFS and OS analyses. Progression information (presence or absence of progression) was based on clinic notes. If the last clinic note date was prior to treatment start, the patient was not eligible for PFS analysis. If the last OS follow-up date was prior to treatment start, the patient was not eligible for OS analysis.  ALT, alanine aminotransferase; ECOG PS, Eastern Cooperative Oncology Group performance status; ; ICI, immune-checkpoint inhibitor therapy; INRb, international normalized ratio; NSCLC, non–small cell lung cancer; PBC, platinum-doublet therapy; rwPFS, real-world progression-free survival; ULN, upper limit of normal. | | | |

### **Supplementary Figure 1.** Distribution of weights in real-world cohorts receiving first-line PBC (A), first-line ICI+PBC (B), or second-line ICI (C)


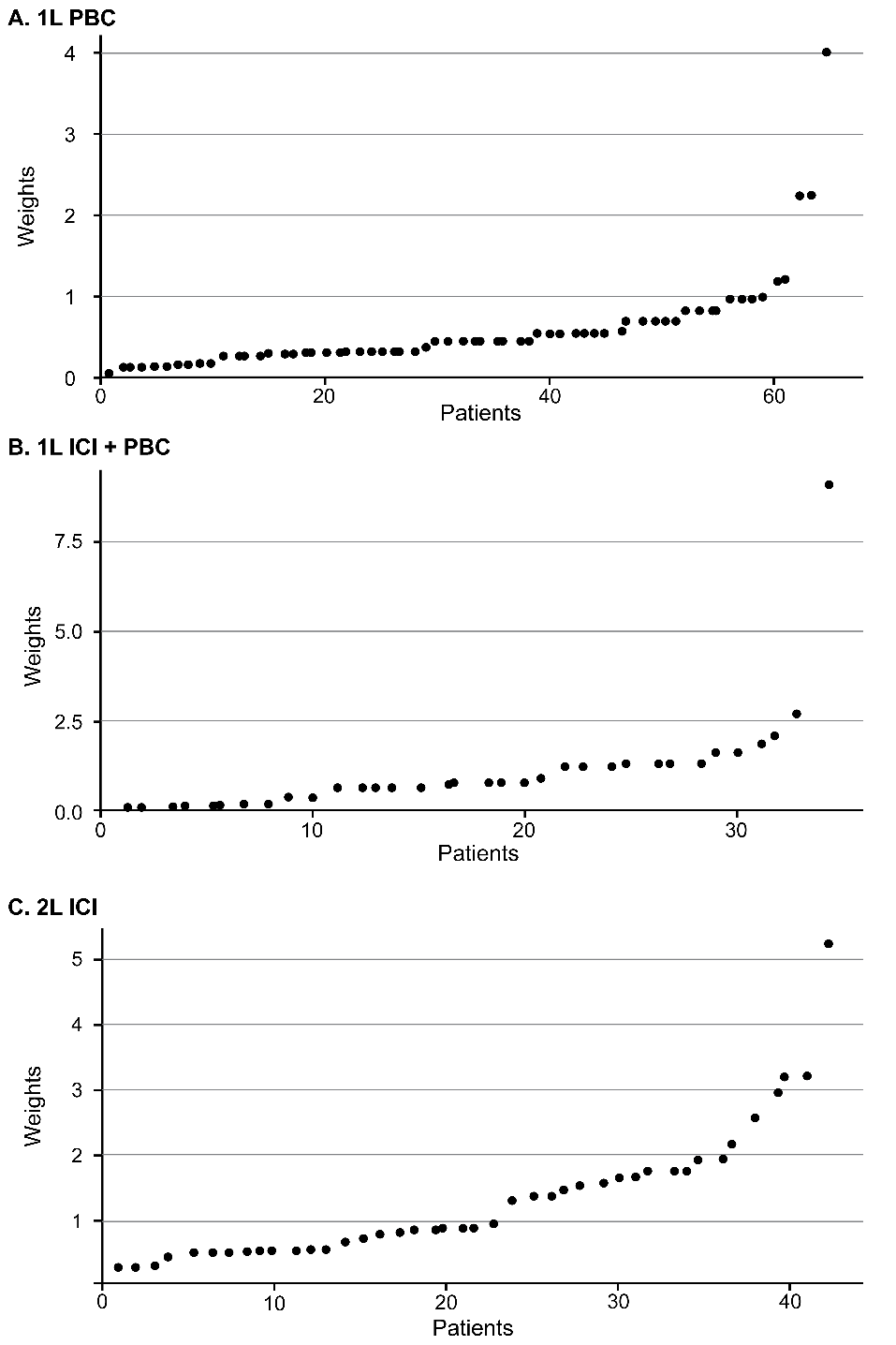


Weights assigned to real-world patients, corresponding to their odds of being in the respective dab-tram cohort, were calculated using the PS, given prespecified baseline covariates, and converted to the odds scale (PS/[1 – PS]).

In the second-line ICI + PBC cohort, 1 patient with weight 9.1 was considered as having an extreme weight and was excluded (trimmed) from the weighted analysis.

ICI, immune-checkpoint inhibitor therapy; PBC, platinum-based chemotherapy; PS, propensity score.

**Supplementary Figure 2.** Standardized mean differences of weighting variables between cohorts before and after weighting by odds

**
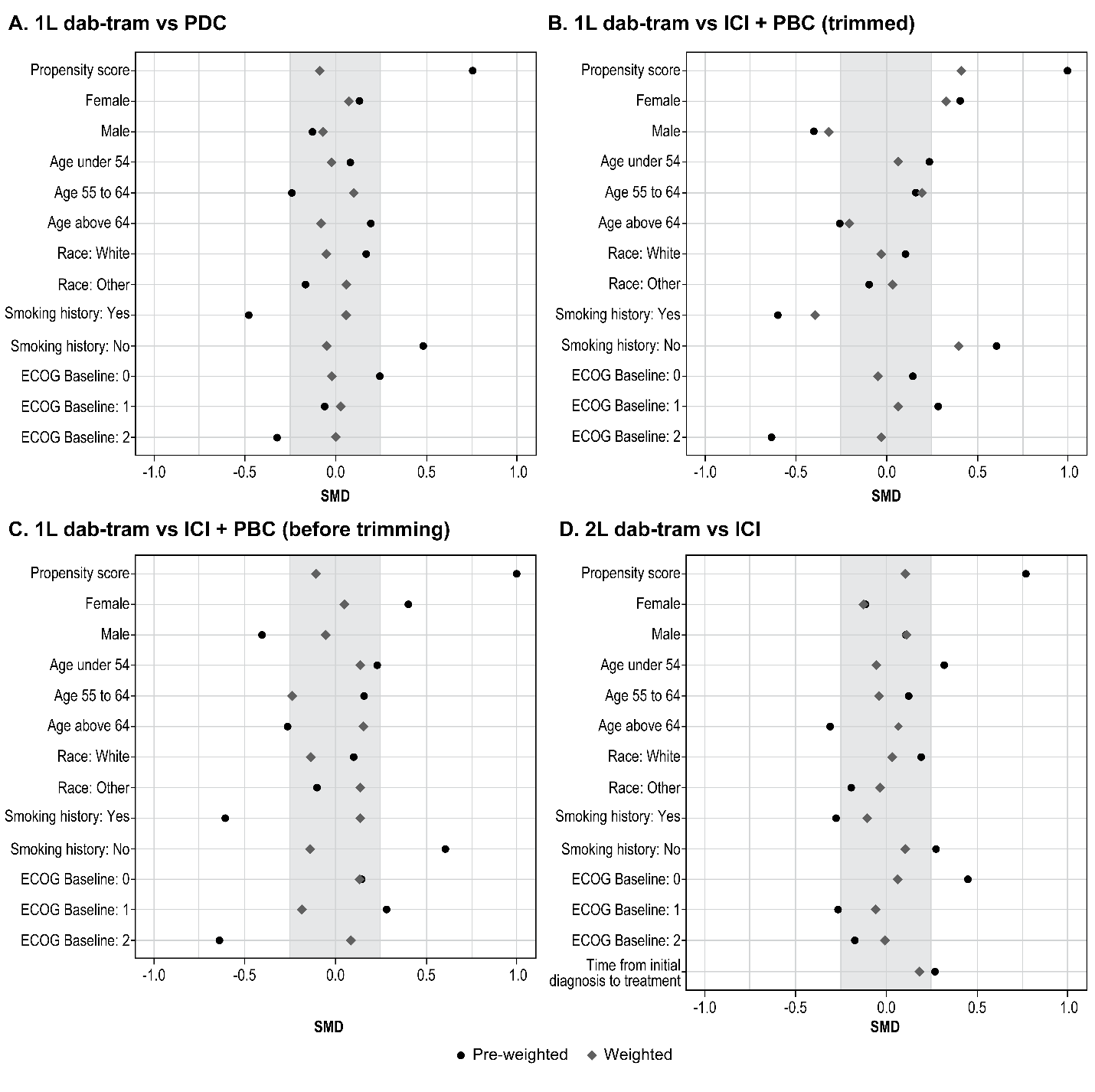
**

SMDs were calculated by dividing the mean differences by the standard deviation of the outcome where the pooled standard deviations were used. Grey areas (SMD ≤0.25) indicate an acceptable range of standardized mean differences. Dots refer to the SMD of weighting variables before weighting by odds. Diamonds refer to the SMD of weighting variables after weighting. Race was recategorized as “White” versus “other,” with “other” including Black, African, Asian, other, and unknown.
ECOG PS, Eastern Cooperative Oncology Group performance status; ICI, immune-checkpoint inhibitor therapy; PBC, platinum-based chemotherapy; SMD, standardized mean difference
